# Rheological, Surface Tension and Conductivity Insights on the Electrospinnability of Poly(lactic-co-glycolic acid)-hyaluronic Acid Solutions and Their Correlations with the Nanofiber Morphological Characteristics

**DOI:** 10.3390/polym14204411

**Published:** 2022-10-19

**Authors:** Ziqian Liu, Seeram Ramakrishna, Ifty Ahmed, Chris Rudd, Xiaoling Liu

**Affiliations:** 1 Department of Mechanical, Materials and Manufacturing Engineering, The University of Nottingham Ningbo China, Ningbo 315100, China; 2 Department of Mechanical Engineering, National University of Singapore, Singapore 119077, Singapore; 3 Department of Mechanical, Materials and Manufacturing Engineering, University of Nottingham, Nottingham NG7 2RD, UK; 4 James Cook University Singapore, Singapore 387380, Singapore

**Keywords:** chain entanglements, electrospinning, solution rheology, solution conductivity, surface tension

## Abstract

In this study, solutions were prepared with fixed concentrations of hyaluronic acid (HA) but varied concentrations of poly (lactic-co-glycolic acid) (PLGA) to emphasize the effects of PLGA concentration and HA addition on solution properties and to further evaluate their electrospinning performance. The dependence of specific viscosity on PLGA concentration was studied to determine the concentration regimes and evaluate the critical concentration (C_e_) for successful fiber generation. The C_e_ of PLGA solutions is 12.07% compared to 10.09% for PLGA-HA solutions. Blending with HA results in a lower concentration dependence and better consistency to the theoretical scaling mechanisms due to the additional topological constrains, which thus result in more chain entanglements. Solutions in semi-dilute entangled regimes show the crossover of complex moduli, verifying the stable and reliable entanglement network. Higher concentrations and HA addition both led to lower crossover frequencies and, thus, a longer relaxation time. The effects of a higher PLGA concentration and HA addition on the surface tension were not evident. However, the HA addition significantly improved the solution conductivity up to three times in the pure PLGA solutions due to its polyelectrolyte nature. Defect-free and uniform nanofibers were generated from 35% to 40% of the PLGA-HA solutions, yet fibers with bead-on-string structures were produced from all studied pure PLGA solutions. Such solution characteristics and parametric correlations can provide predictive insights on tailoring the morphological characteristics of nanofibers for specific applications.

## 1. Introduction

Electrospinning is a simple and versatile process to fabricate micrometer and nanometer scale fibers and shows great potential in various applications such as tissue engineering, water treatment, energy generation and storage, electronics, and wearables [1]. Generally, a highly electrified polymer solution or melt undergoes a whipping motion to stretch and elongate into fibers. Upon electrification, a pendant droplet is initially generated at the tip of the spinneret and deforms into a Taylor cone due to the electrostatic repulsion among the surface charges [2]. Once the strength of the electric field yields a critical level, a charged jet is ejected from the Taylor cone, and undergoes the whipping motion before finally solidifying into fibers upon solvent evaporation. There are a variety of forces governing the electrospinning process, including the electrostatic force, the viscoelastic force, the surface tension force, the gravity force, and the air drag force due to air friction [3]. Parameters that control the electrospinning process mainly alter such force balance and aid in solvent evaporation. These include process parameters such as the applied electric field, the feeding rate, the distance between the capillary and collector, ambient conditions, and the solution [2].

The inherent properties of solutions, including solution viscosity, conductivity, and surface tension, play a dominant role on their electrospinnability [4]. Solution viscosity, as a measure of polymer chain entanglements, is critical on the generation and morphology of electrospun fibers [5]. This is because an insufficient chain entanglement would lead to the jet break-up and droplet formation termed as electrospraying. It has been verified by numerous studies that a higher solution concentration leads to an increased viscosity and less beads on electrospun fibers [6]. In addition, there is a minimum polymer concentration known as the critical entanglement concentration (C_e_), which is required for successful fiber generation [7,8]. This necessitates a detailed investigation of the dependence of fiber morphology on solution rheological behavior. The rheological properties of polymer solutions can offer a critical clue to determine the optimum processing conditions in the electrospinning process [9]. Researchers investigated the concentration dependence of viscosity with scaling mechanisms and identified four different concentration regimes, including dilute, semi-dilute unentangled, semi-dilute entangled, and concentrated regimes in good solvents (interactions between the polymer segments and solvent molecules are energetically favorable), and theta solvents (polymer–polymer self-interactions are preferred) as summarized in Table 1 [10,11,12,13]. For solutions in the dilute regime, polymer chains are isolated by solvent molecules, which leads to an unsuccessful electrospinning process. They start to overlap each other in the semi-dilute unentangled regime, whereas the entanglement remains insufficient to afford successful electrospinning. As the concentration increases, the semi-dilute, unentangled regime transits to a semi-dilute entangled regime marked by the C_e,_ where beaded but continuous fibers are produced.

When the polymer concentration is above C_e_-enabling, sufficient chain entanglements for fiber generation, the balance between the solution viscosity, surface tension, and solution conductivity control the electrospun fiber morphology. In the electrospinning process, the stretching effect by an electrostatic force is mainly determined by the external applied electric field and the charge distribution among the jet. Solution conductivity plays a critical role in charge density and distribution. It is reported that more conductive solutions are prone to yield more uniform fibers and allow for a lower minimum voltage for electrospinning [3,14]. By contrast, the viscoelastic force due to the viscosity of the solution and the surface tension force counterbalances the stretching force and resists it. Rheological insights on the electrospinnability of polymer solutions have been widely studied by focusing on the polymer chain structure and solvent composition [4,7,15,16]. However, systematic investigations on the electrospinnability of solutions, which are multi-component in terms of rheological behavior, conductivity, and surface tension, are lacking. Furthermore, past rheological studies have mainly focused on the relationship between the viscosity and electrospinnability of polymer solutions by investigating their concentration dependence and C_e_. Other critical rheological factors such as the elastic (G′) and viscous (G″) moduli and their relationship are also studied with a focus on the different solvent systems and different polymer concentrations [7,8,17]. Herein this article, we studied the dependence of electrospun fiber morphology on those solution properties of a dual-polymer solution system. The relationship between the solution concentration and viscosity was analyzed to determine the critical C_e_. Meanwhile, complex moduli in rheological aspects were studied to verify the corresponding C_e_.

Since the first electrospun product as a wound dressing mat was patented in 1977 [18], electrospinning has been widely explored for healthcare applications. The relatively large surface-to-volume ratio, high porosity, and tailorable pore size of nanofibrous structure give electrospun films the potential for use in tissue engineering and regenerative medicine, drug delivery, biosensors, diagnostics, etc. [1]. PLGA has been widely used in biomedical applications such as tissue engineering and drug delivery systems with FDA approval owing to its excellent biocompatibility and controllable biodegradation rate [19]. However, the hydrophobicity and intermediate mechanical strength of PLGA can limit its applications [20]. Extensive studies focus on the incorporation of other components such as collagen [21], gelatin [22], chitosan [23], cellulose nanocrystals [20], multi-wall carbon nanotubes [24,25], and nano-hydroxyapatite [26] into the electrospinning system of PLGA to enhance its properties and benefit its applications in specific tissues. The effects of solvent type and solvent composition on the electrospinning of pure PLGA solutions are studied by Liu et al. [15]. However, there is a lack of study on how additional components affect the electrospinnability of PLGA blending solutions. Hyaluronic acid (HA), as a naturally occurring glycosaminoglycan with a high molecular weight, is commonly found in connective tissue and body fluids [27]. It plays a critical role in all stages of inflammation and wound healing due to its excellent viscoelastic, rheological, and hygroscopic characteristics [28]. This paper aims to study the relationship between PLGA-HA solution properties and their electrospinnability by emphasizing the effects of PLGA concentration and HA addition. The mechanisms underlying the fiber formation and factors governing the fiber morphology in the electrospinning process of a dual-polymer system are investigated.

## 2. Materials and Methods

### 2.1. Materials

PLGA ((75:25), Mw = 66,000–107,000 Da), hyaluronic acid (sodium salt from Streptococcus equi, Mw = 403.31 (monomer), 800 KDa~1.0 MDa), and dimethyl sulfoxide (DMSO) were purchased from Shanghai Aladdin^®^ Biochemical Technology (Shanghai, China) and used as received.

### 2.2. Rheology Study

Solution rheology was conducted using a Kinexus rheometer (Malvern, UK) at 25 °C with the cone-and-plate geometry (cone angle: 1°; cone radius: 25 mm) for homogenous shear conditions. Shear rate sweeps from 0.1 to 1000 s^−1^ were conducted to determine the shear viscosity of each solution. Oscillatory strain sweep tests were performed first to determine the linear viscoelastic region of each solution. Oscillatory frequency sweep tests were performed within the linear viscoelastic region from 0.1 to100 Hz at a shear strain of 0.5%.

### 2.3. Solutions Preparation and Electrospinning Process

Pure PLGA solutions were prepared by dissolving PLGA in DMSO at varied concentrations of 2%, 5%, 8%, 10%, 15%, 18%, 20%, 22%, 25%, 28%, 30%, 35%, and 40% (*w/v*). PLGA-HA solutions were prepared by adding HA to a fixed concentration of 1.5% (*w/v*) in the prescribed pure PLGA solutions. The polymer was mixed with the solvent under magnetic stirring at room temperature until fully dissolved.

The electrospinning solutions were fed into a syringe at a flow rate of 10 μL/min with an applied voltage of 20 kV, as described elsewhere [29,30,31]. An aluminum foil covered plate was used as the flat collector at a tip-to-collector distance of 15 cm.

### 2.4. Surface Tension and Solution Conductivity

The surface tension of polymer solutions was investigated using the BZY-2 surface tension meter (Nanbei Instrument Ltd., Zhengzhou, China). Solution conductivity was determined using a conductivity meter (Mettler Toledo, Columbus, OH, USA). Five measurements were taken for each solution.

### 2.5. Scanning Electron Microscopy (SEM) Analysis

Electrospun nanofiber morphology was characterized using SEM (Zeiss, Jena, Germany) at a 3 kV acceleration voltage. Electrospun films were sputter coated with gold (Leica SCD 500 Gold Painter, Leica, Wetzlar, Germany) under an argon atmosphere prior to SEM analysis. For fibers electrospun from each solution, 150 fibers were randomly selected from SEM images for diameter evaluation. The diameter of electrospun nanofibers was measured using ImageJ v1.52a.

### 2.6. Fourier Transform Infrared Spectroscopy (FTIR)

The FTIR spectra of pristine PLGA, pristine HA, and electrospun PLGA-HA films were recorded in attenuated total reflection mode (ATR) by the Nicolet iS FTIR spectrometer (Thermo Scientific, Waltham, MA, USA) equipped with an iD7 diamond ATR crystal. The spectrum was obtained in the range of 500–4000 cm^−1^ with a resolution of 0.482 cm^−1^ and 16 times scanning.

### 2.7. Statistical Analysis

A one-way ANOVA with Tukey’s post-hoc test was used for pairwise comparisons using OriginPro 2018 software. Differences between groups with *p*-values < 0.05 were considered statistically significant. Detailed statistical analysis of solution conductivity, surface tension, and the diameter of electrospun nanofibers between groups with *p*-values is shown in Supplementary Materials.

## 3. Results and Discussion

### 3.1. Solution Rheology and Concentration Regimes for PLGA and PLGA-HA Solutions

#### 3.1.1. Study on Solution Viscosity and Critical Chain Entanglement Concentration

**Figure 1:**
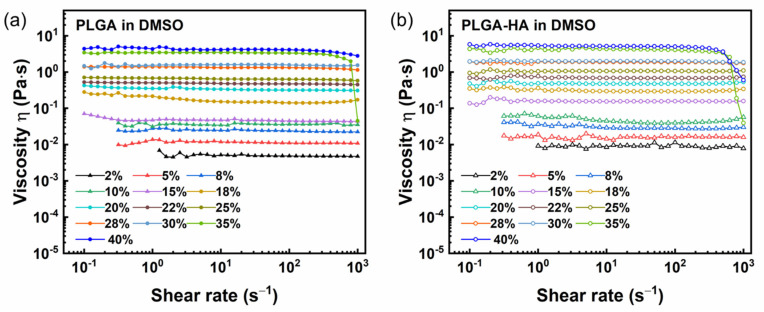
Dependence of solution viscosity on shear rate for (**a**) PLGA in DMSO solutions and (**b**) PLGA-1.5% HA in DMSO solutions.

**Figure 2:**
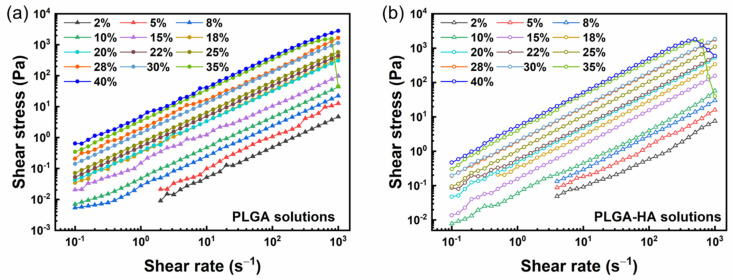
Dependence of shear stress on shear rate for (**a**) PLGA in DMSO solutions and (**b**) PLGA-1.5% HA in DMSO solutions.

The rheological behavior of PLGA and PLGA-HA solutions was investigated on a wide range of PLGA concentrations. The relationship between the solution viscosity and shear rate is studied via shear rate sweep tests and is shown in Figure 1. Generally, both solutions showed Newtonian behavior with plateaued viscosity. Similarly, the linear relationship between the shear stress and shear rate for pure and binary solutions also verified the Newtonian behavior (Figure 2). An increasing PLGA concentration can lead to higher solution viscosity due to the increased molecular weight (Mw) and, thus, more chain entanglements. The value of shear viscosity of PLGA-HA solutions is overall higher compared to those of corresponding pure PLGA solutions. This may be due to the HA addition, which resulted in a higher average Mw and molecular interactions contributing to the increased solution viscosity. It was found that hyaluronan would form a secondary structure involving hydrogen bonds between adjacent sugar units in DMSO, leading to strong intramolecular interactions of HA [32,33]. Such secondary structures contribute significantly to the stability and chain stiffness of the polymer [34]. Shear thinning was observed in between 35% and 40% of PLGA and PLGA-HA solutions at high shear rates (above 500 s^−1^), which could be attributed to shear-induced molecular disentanglements. The similar shear thinning behavior of pure PLGA solutions with a concentration of 30% (*w/v*) at a high shear rate was reported before [15]. Such shear thinning behavior at high shear rates of polymer solutions with high concentrations was also observed in nylon-6,6 and sulfonated polystyrene solutions [35,36]. The shear thinning behavior shows a progressive breakdown of the intermolecular network, which is more pronounced in concentrated solutions compared to dilute solutions [33,37].

It is noted that the viscosity values in both kinds of solutions can be categorized into two ranges which are characterized by a sharp increase. The first range for PLGA solutions is 2–15% with a viscosity lower than 0.05 Pa∙s; the other one is 18–40% with a range of 0.15–4.21 Pa∙s. For PLGA-HA solutions, the two ranges are 2–10% with a viscosity lower than 0.05 Pa∙s and 15–40% with a range of 0.29–5.21 Pa∙s. This suggests that there are two different concentration regimes in both solutions. Here, the flow curves in Figure 1 were fitted to the Cross model to extrapolate the zero-shear rate viscosity of each solution as shown below [38]:

$$\eta =\eta_{\infty }+\frac{\eta_{0}-\eta_{\infty }}{1+{\left(kx\right)}^{n}}$$

The specific viscosity can be calculated from the equation as follows:

$$\eta_{sp}=\frac{\eta_{0}-\eta_{s}}{\eta_{s}}$$

where $\eta_{\infty }$ = infinite viscosity, $\eta_{0}$ = zero-shear rate viscosity,$\;\eta_{s}$ = solvent viscosity, *x* = shear rate, and *k* and *n* are constants.

**Figure 3:**
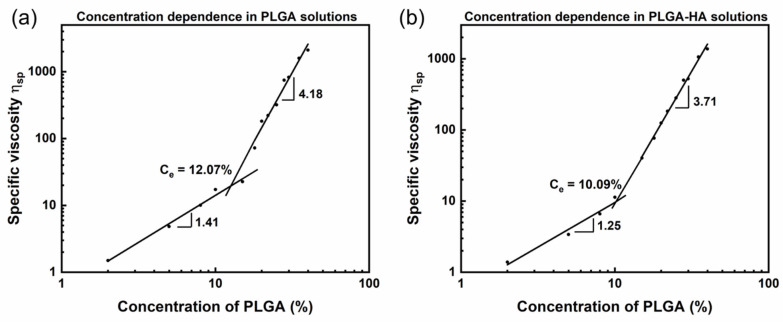
Dependence of specific viscosity on PLGA concentration for (**a**) PLGA in DMSO solutions, and (**b**) PLGA-1.5% HA in DMSO solutions.

The dependence of a specific viscosity on PLGA concentrations for two kinds of solutions is shown in Figure 3. For pure PLGA solutions at low concentrations (C_PLGA_ < 18%), the scaling $\eta_{sp}$~C^1.41^ is in agreement with the scaling mechanism in the semi-dilute unentangled regime. As the concentration increased, the $\eta_{sp}$~C^4.18^ marked the onset of a semi-dilute entangled regime, which is in line with the scaling theory of good solvents. The entanglement concentration C_e_ in pure PLGA solutions was extrapolated from the transition from semi-dilute unentangled to semi-dilute entangled regions with a value of 12.07%. By contrast, the scaling predictions in PLGA-HA solutions showed a lower concentration dependence on the viscosity and better consistency with the theoretical values ($\eta_{sp}$~C^1.25^ in semi-dilute unentangled and $\eta_{sp}$~C^3.75^ in semi-dilute entangled). The specific viscosity of PLGA-HA solutions was scaled with $\eta_{sp}$~C^1.25^ in semi-dilute unentangled regions and $\eta_{sp}$~C^3.71^ in semi-dilute entangled regimes. The critical entanglement concentration C_e_ in PLGA-HA solutions is 10.09%, which is lower than that of PLGA solutions suggesting its better potential performance in the electrospinning process at the same concentration.

The addition of HA slightly mitigated the concentration dependence, as verified by lower scaling exponents and the lower entanglement concentration of PLGA-HA solutions compared to pure PLGA solutions. Such an effect is more marked in semi-dilute, unentangled regimes. The introduction of HA to the solution led to a higher Mw, which occupied a larger hydrodynamic volume. Polymer chains in a high Mw allow sufficient topological constraints at a lower concentration to realize chain entanglements. Therefore, the C_e_ of PLGA-HA solutions can be lower than that of PLGA solutions. Besides the elevated Mw, extra molecular interactions due to the HA addition can also account for the lower concentration dependence. Mckee et al. found that the branching structure of the polymer can lead to a much weaker concentration dependence in semi-dilute entangled regimes and a higher C_e_ than linear structures due to the hindered effect of the branch points on chain overlap [4,39]. Casasola et al. studied a binary-solvent system for PLA and reported the solution viscosity in semi-dilute unentangled region scales with *η*~C^0.66^ showing a relatively weaker concentration dependence due to the strong effect of solvent interactions on the solution rheology [17]. In our study, the lower dependence could possibly be owing to the interaction between HA molecules, solvents, and their intramolecular interactions. It was found that the rheology of HA solutions is typical of polyelectrolytes, and there are electrostatic interactions in polyelectrolyte solutions with unclear effects on topological constraints [40,41]. It suggests that an extended conformation occurs in salt-free polyelectrolyte solutions due to the electrostatic repulsion between the charged groups. Such an extended structure may benefit adjacent chain interactions and result in potential chain entanglements at a lower concentration.

#### 3.1.2. Study on Complex Moduli and Their Crossover Frequency

**Figure 4:**
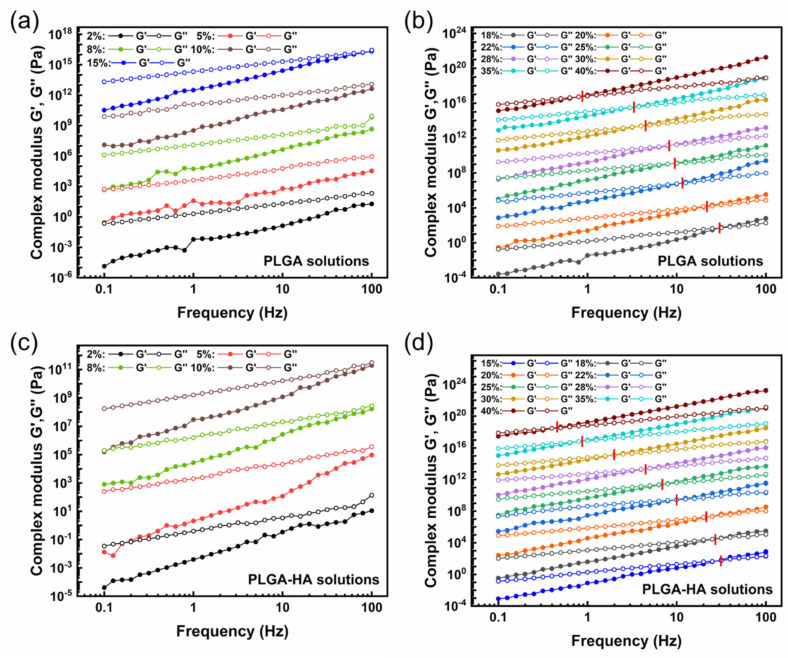
Crossover of complex moduli for different PLGA concentrations of pure PLGA solutions (**a**,**b**) and PLGA-1.5% HA solutions (**c**,**d**). (**a**,**c**) showed no crossover point for G′ and G″ in unentangled regimes for both solutions; however, (**b**,**d**) showed the corresponding crossover frequency for G′ and G″ as marked by the red lines. The curves were shifted with y-displacement to visually show the crossover point. Curves of complex modulus without shifting are presented in Supporting Information Figure S1. The Cox–Merz rule was validated and is shown in supporting information Figure S2.

For solutions in the semi-dilute unentangled regime (C_PLGA_ ≤ 15% for PLGA solutions and C_PLGA_ ≤ 10% for PLGA-HA solutions), the loss modulus (G″) dominated the storage modulus (G′) for the entire tested frequency resulting in no crossover between G′ and G″ (Figure 4a,c). Whereas the G″ is only higher than G′ at a low frequency for solutions in the semi-dilute entangled regime (C_PLGA_ ≥ 18% for PLGA solutions and C_PLGA_ ≥ 15% for PLGA-HA solutions), both G′ and G″ increased with a higher frequency. However, G′ showed a higher increasing rate and eventually dominated G″ resulting in the crossover of G′ and G″. A higher concentration led to an increased number of molecular interactions, and as a result, the polymer chains started to entangle, leading to the equivalent relationship between G′ and G″ as marked by the crossover point.

The crossover of G′ and G″ is another critical characteristic on solution electrospinnability related to the existence of stable and sufficient entanglements. The viscoelastic properties of polymer solutions are dominated by the presence of entanglement networks or their equivalent topological constraints [42]. The absence of crossover points suggests the instability of the possible polymer network in those solutions [43]. There might be a temporary network with flexible connectors, and the potential entanglement slippage under stress may be due to a low concentration and low Mw [5]. In contrast, a more stable and reliable entanglement network was detected in solutions on the semi-dilute entangled regime exhibiting crossover points [42]. Before the crossover point where G″ was higher than G′, the solution showed liquid-like behavior with irreversible energy loss. After the crossover point where G′ was larger than G″, solutions showed more elastic behavior and exhibited the ability to reversibly store the energy. Such dominant elastic characters of solutions after the crossover point would benefit fiber production in the electrospinning process [7].

It is worth noting that the frequency at which G′ crossover with G″ decreased with an increase in PLGA concentrations, as Figure 4b,d shows. Additionally, PLGA-HA solutions exhibited a lower crossover frequency than PLGA solutions at the same PLGA concentration. Generally, the double logarithmic plot of complex moduli versus frequency consists of three zones: terminal, plateau, and transition zones in a three-zone model [44]. The crossover points of G′ and G″ at higher frequencies marked the transition zone, whereas the crossover point at lower frequencies marked the transition of the terminal zone (viscous) to the rubbery-plateau zone (elastic) [45]. Those two crossover points delimit the emergence of a plateau zone and indicate the entanglement network. Two sets of characteristic relaxation times can also be determined by the crossover frequency in the three-zone model: the long-range relaxation time λ_1_ in the terminal zone and the short-range relaxation λ_2_ in the transition zone. The crossover frequency is inversely proportional to the relaxation time. Only a long-range relaxation time was determined in our study. Solutions with a higher PLGA concentration and HA addition exhibited a lower crossover frequency indicating their longer relaxation time. The relaxation time implies the configurational rearrangements of the global polymer structure in response to the applied force between entanglements (λ_1_) and beyond entanglements (λ_2_) [42]. Solutions in the semi-dilute entangled regime exhibited higher viscosity and larger Mw. Polymer chains in those solutions thus showed lower mobility and a higher entanglement degree. It required a longer period to reorient to their initial state after the shear force was removed. Similarly, PLGA-HA solutions showed a higher viscosity and larger local structures than the PLGA solutions resulting in longer relaxation times. Longer relaxation times indicate the limited chain motions of a larger scale, which is related to the elastic character of solutions and benefits of fiber generation [7].

### 3.2. Surface Tension and Solution Conductivity

**Figure 5:**
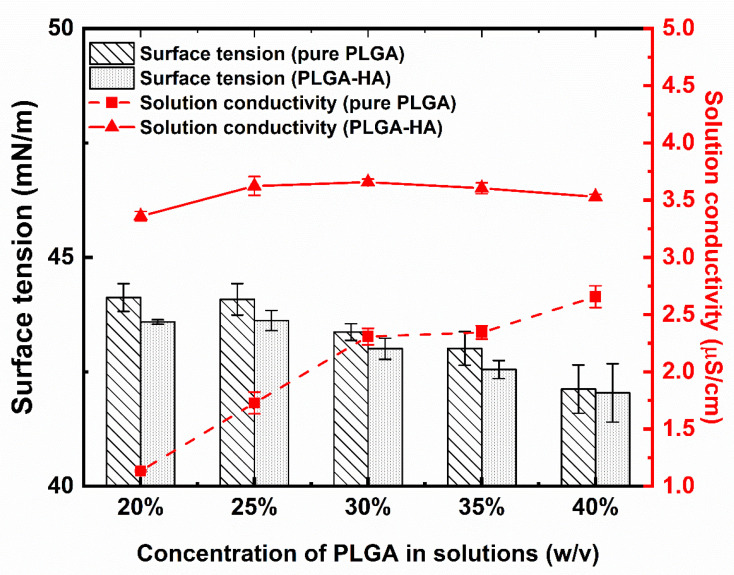
Solution properties of PLGA in DMSO and PLGA-1.5% HA in DMSO solutions. *n* = 5.

The results of the solution properties, including solution viscosity, solution conductivity, and surface tension for both pure PLGA solutions and PLGA-HA solutions, are presented in Figure 5. Here, only solutions with concentrations higher than 20% are included. The statistical analysis of solution conductivity and surface tension with *p*-values for those two kinds of solutions is summarized in Supplemental Table S1 and Table S2, respectively.

Generally, both the solution viscosity and conductivity of PLGA-HA solutions were higher than those of pure PLGA solutions at the same concentration. For the pure PLGA solution, the values of solution conductivity increased with the PLGA concentration and ranged from 1.13 ± 0.01 to 2.66 ± 0.10 μS/cm. Fong et al. also found that the solution conductivity increased as the concentration of PEO increased [6]. The value of the solution conductivity of PLGA-HA solutions ranged from 3.36 ± 0.04 to 3.66 ± 0.03 μS/cm. The HA-incorporated solutions showed significantly (*p* < 0.001) higher solution conductivity values than those pure PLGA solutions with the same PLGA concentration. In addition, the solution conductivity of PLGA-HA solutions showed slighter variance compared to the pure PLGA solutions, which suggests that the effect of PLGA concentration on solution conductivity is less pronounced compared to HA addition. This may be due to the polyionic repeating groups in HA, which would dissociate and become ionized when dissolved in polar solvents, thus leading to charged polymer chains together with the releasing of counterions [46]. Therefore, the incorporation of HA in PLGA solutions resulted in a more charged state and a predictable higher solution conductivity. The overall surface tension of the PLGA-HA solutions was lower than that of the pure PLGA solutions. Generally, the surface tension of solutions decreased with an increased PLGA concentration for both kinds of solutions. The surface tension of pure PLGA and PLGA-HA solutions were reduced to a comparable level when the concentration increased to 40%. The effects of HA addition on the reduction in surface tension were less pronounced than increasing PLGA concentrations.

The surface tension of semi-dilute polymer solutions in good solvent conditions was once examined both experimentally and theoretically by di Meglio et al. [47]. The contact between a solid-liquid or a liquid–gas interface was considered by introducing a wall into the system. Generally, the surface tension was treated in terms of the Gibbs adsorption equation [48]. Di Meglio et al. generated the interfacial energy γ following the Cahn development for interfacial energies and wetting, as shown below [49]:

(1) $$\gamma -\gamma_{0}=\gamma_{1}\Phi_{s}+\int_{0}^{\infty }dz\left\{F\left(\Phi \right)-\mu_{b}\Phi +\Pi_{b}+L\left(\Phi \right){\left(\frac{d\Phi }{dz}\right)}^{2}\right\}\;$$

In Equation (1), $\gamma_{0}$ is the surface tension of the pure solvent, $\gamma_{1}$ is a local solute-interface interaction energy/area, the z-direction is perpendicular to the surface and measured into the solution, F(Φ) is the free energy density for a semi-dilute polymer solution of volume fraction Φ, $\mu_{b}$ is the chemical potential, $\Pi_{b}$ is the bulk osmotic pressure, L(Φ) describes the solution stiffness to spatial fluctuations of the concentration, and $\Phi_{s}$ is the solute volume fraction. After a series of assumptions and analyses based on the mean field theory, they presented a linear variation in the surface tension in Φ for a good solvent:

(2) $$\gamma -\gamma_{0}≅\gamma_{1}\Phi_{b}\;$$

In Equation (2), $\gamma_{1}<0$ for the attractive walls and $\gamma_{1}>0$ for the repulsive walls. Such a linear form of the bulk concentration ($\Phi_{b}$) dependence of surface tensions can provide insights on the results of our study. Increasing PLGA concentrations and the addition of HA both decreased the surface tension of polymer solutions. This also suggests the attractive interface between DMSO and PLGA [50].

### 3.3. FTIR Spectroscopy

**Figure 6:**
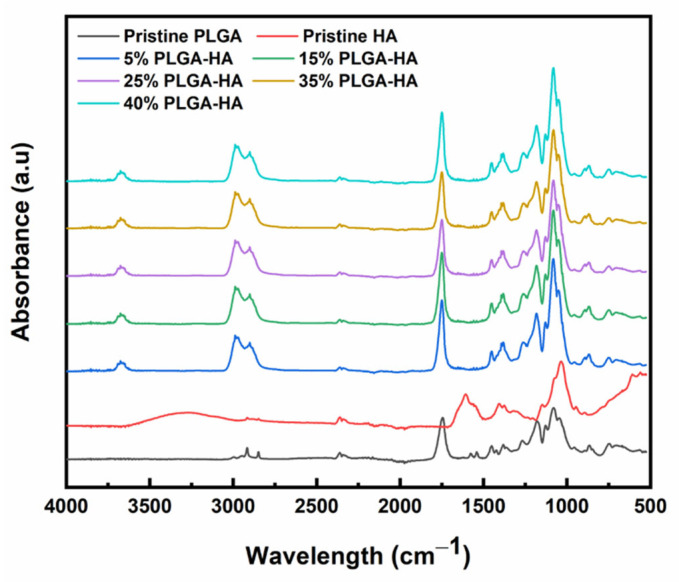
FTIR spectra of pristine PLGA, HA, and electrospun PLGA-HA films.

The ATR-FTIR spectra of pristine PLGA, pristine HA, and electrospun PLGA-HA films are shown in Figure 6. The characteristic peaks of PLGA, including an ester carbonyl stretching around 1747 cm^−1^ (C=O), C-O-C symmetric stretching around 1082 cm^−1^, and other methylene and methyl groups (CH, CH_2_, CH_3_) at 2840–3035 cm^−1^ were observed in both pristine PLGA and electrospun PLGA-HA films [51,52]. The spectra of pristine HA exhibits OH and NH stretching at 2974–3587 cm^−1^, secondary amide groups at 1606 cm^−1^, C-O group combined with C=O around 1406 cm^−1^, and C-O-C stretching at 1033 cm^−1^ [53,54,55]. The peaks around 3600 cm^−1^ in electrospun PLGA-HA films could be attributed to the addition of HA in the solutions. It was reported that the frequency shift in the spectroscopy was induced by molecular interactions, and such a magnitude is correlated to the strength of hydrogen bonding [56]. The OH stretching is sensitive to hydrogen bonding interactions [57]. Here the shift of the OH stretching peak of HA indicates a weakening effect on the hydrogen bonds [58]. During solution preparation, HA in DMSO would form a secondary structure involving hydrogen bonds [32]. Upon processing and solvent evaporation, such hydrogen bonding and OH stretching could be weakened in PLGA-HA films compared to pristine HA powders. This led to the peak shift in OH stretching to a higher wavenumber (~3600 cm^−1^). Other characteristic peaks of HA were not apparent due to the overlapping common peaks, such as C=O and C-O-C stretching [26]. Overall, the FTIR results can indicate the existence of PLGA and HA in the electrospun films and confirm the binary components in electrospinning PLGA-HA solutions.

### 3.4. Electrospun Nanofibers Characterisation

**Figure 7:**
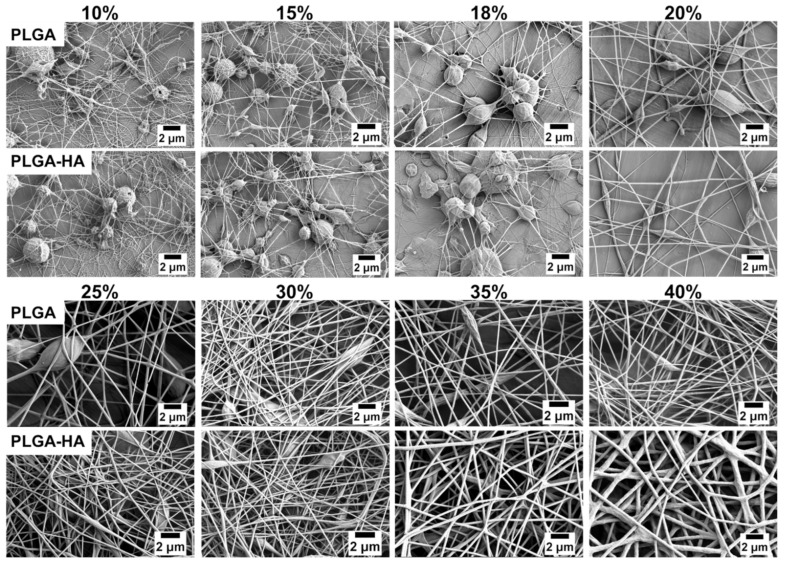
SEM results of electrospun nanofibers generated from pure PLGA in DMSO solutions and PLGA-1.5% HA in DMSO solutions with different concentrations of PLGA.

To evaluate the electrospinnability of each solution, electrospun samples were characterized via SEM, with the results shown in Figure 7. In theory, solutions with polymer concentrations higher than C_e_ would result in successful electrospinning with generations of continuous fibers. It was found that for solutions with a concentration between 10 and 20%, which exceeded the C_e_, fragmented fibers with aggregated and circular beads were generated. Continuous fibers yet with detectable beads were generated in 25% PLGA solutions and 20% PLGA-HA solutions. It has been investigated that bead-less fibers could be generated with a concentration of 2–2.5 times C_e_ [4,5,7,8]. Uniform and defect-free fibers were produced in between 35% and 40% of PLGA-HA solutions in which the PLGA concentration was approximately three times that of C_e_. On the contrary, there were still a few beads observed in 35% PLGA and 40% PLGA nanofibers. Possible reasons for the underestimation of the defect-free concentration could be the assignable effects of the surface tension and solution conductivity on the fiber morphology. The solution viscosity plays a critical role in fiber morphology via control over chain entanglements. Once the concentration of PLGA increased to exhibit a critical viscosity and chain entanglements, the electrospinnability of the solutions was also synergistically affected by the surface tension and solution conductivity.

Solutions with higher PLGA concentrations and HA addition generally produced fibers with a more uniform and defect-free structure, which could be attributed to their higher conductivity and lower surface tension. The beads in PLGA-HA nanofibers were more elongated with a spindle-shape, whereas the beads in pure PLGA nanofibers at the same concentration showed a more circular morphology. Electrospinning solutions with a higher surface tension were prone to generate bead-on-string structures. Because surface tension favors a lower value of surface area per unit mass, this would force jets into a spherical shape [6]. Since each solution was subjected to the same applied potential, a higher solution conductivity would allow for a better charge distribution along the surface, leading to a more smooth and uniform fiber morphology. The viscoelastic force due to viscosity and the surface tension force was overcome by the applied electrostatic force to generate fibers [3]. Therefore, solutions with a higher conductivity and lower surface tension were prone to generate more uniform and elongated defect-free structures.

**Figure 8:**
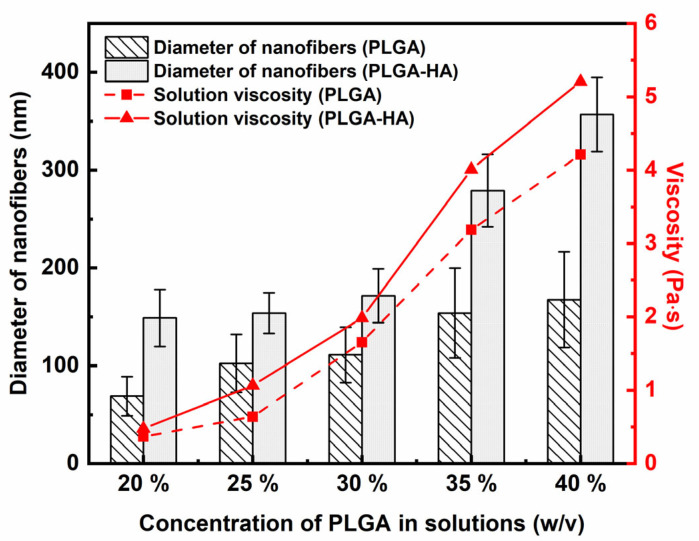
Solution viscosity of electrospinning solutions at different concentrations, and diameter evaluation of their corresponding electrospun nanofibers. *n* = 150.

The diameter of electrospun fibers generated from critical concentrations was evaluated and is presented in Figure 8. A detailed statistical analysis of the diameter of electrospun fibers with *p*-values for those two kinds of solutions is summarized in Table S3. It was found that solutions with higher concentrations of PLGA produced thicker nanofibers. Higher polymer concentrations resulted in larger solution viscosity, suggesting that more entanglement couplings led to thicker electrospun nanofibers [4]. Similarly, electrospun fibers from PLGA-HA solutions exhibited significantly thicker (*p* < 0.001) diameters than those from PLGA solutions at the same concentration due to the relatively higher viscosity of PLGA-HA solutions than pure PLGA solutions. Although a different PLGA content and HA addition can lead to varied solution conductivity and surface tension, the variance in solution viscosity was more notable. The viscosity of those two kinds of solutions varied across an order of magnitude with increased concentrations (Figure 8). It suggests the dominant role of solution viscosity on the diameter of electrospun nanofibers over surface tension and solution conductivity, which is also observed by other studies. Casasola et al. [17] reported that PLA solutions with a higher concentration showed a relatively higher solution conductivity and lower surface tension. However, the produced electrospun nanofibers exhibited a considerably larger diameter due to the significantly higher solution viscosity. Liu et al. also found that viscosity played a dominant role in fiber generation even though surface tension and viscosity both varied with PLGA concentrations [15].

## 4. Conclusions

Overall, we studied the relationship between the properties of PLGA and PLGA-HA solutions and their electrospinnability. Understanding the solution characteristics can provide predictive insights into the success of the electrospinning process. Generally, the shear viscosity increased with increased PLGA concentrations and the incorporation of HA due to higher Mw and chain entanglements. Semi-dilute unentangled and semi-dilute entangled concentration regimes were detected in both solutions. The scaling predictions between the PLGA concentration and the specific viscosity were different in those two kinds of solutions, yet both were consistent with theoretical values. PLGA-HA solutions showed lower concentration dependence with a lower C_e_ of 10.09% than PLGA solutions with a C_e_ of 12.07% due to high Mw and the possible extended conformation of HA in solutions. The crossover between G′ and G″ detected was only found in semi-dilute entangled solutions confirming the sufficient and stable entanglement network. The network can withstand stress and store elastic energy, which favors fiber generation instead of droplets in the electrospinning process.

Increasing the concentration of PLGA and blending with HA can enhance the solution conductivity together with decreasing the surface tension. We found that the defect-free concentrations of PLGA-HA solutions are around three times the C_e_ (~35%). Viscosity shows a dominant role in the diameter of electrospun fibers over surface tension and solution conductivity. The control over PLGA concentration on solution properties is mainly attributed to the change in Mw to affect chain entanglements. However, blending with HA results is a more complex condition where the additional molecular interactions, the conformational change of HA in solvents, the polyelectrolyte nature, and the relatively high Mw of HA should all be considered. This suggests that the electrospinning system with blending components should be thoroughly investigated to obtain optimal electrospun products in the future.

## Figures and Tables

**Table 1:** Summary of scaling predictions between concentration and $\eta_{sp}$ (specific viscosity) in different concentration regimes.

Concentration Regime	Scaling Predictions	
	Good Solvent	Theta Solvent
Dilute	$\eta_{sp}\sim C$	
Semi-dilute unentangled	$\eta_{sp}\sim C^{1.25}$	$\eta_{sp}\sim C^{2}$
Semi-dilute entangled	$\eta_{sp}\sim C^{3.75}$	$\eta_{sp}\sim C^{4.68}$
Concentrated	$\eta_{sp}\sim C^{3.6}$	

## Data Availability

Not applicable.

## Supplementary Materials

The following supporting information can be downloaded at: https://www.mdpi.com/article/10.3390/polym14204411/s1, Figure S1: Complex moduli for PLGA and PLGA-HA solutions. Generally, higher PLGA concentration shows higher module values. Figure S2: Cox-Merz relationship between steady shear viscosity (filled symbols) and complex viscosity from oscillatory experiments (open symbols) and for PLGA and PLGA-HA solutions of selected concentrations (5%, 10%,15%, 20%, 25%,30%, 35% and 40%); Table S1: Statistical analysis of solution conductivity results with *p*-values investigated. Table S2: Statistical analysis of solution surface tension results with *p*-values investigated. Table S3: Statistical analysis of diameter of electrospun nanofibers with *p*-values investigated. Reference [59] is cited in the Supplementary Materials.
