# Supplementary material for: Rheological, Surface Tension and Conductivity Insights on the Electrospinnability of Poly(lactic-co-glycolic acid)-hyaluronic Acid Solutions and Their Correlations with the Nanofiber Morphological Characteristics
Source: Polymers (Basel). 2022 Oct 19;14(20):4411. doi: 10.3390/polym14204411 (PMC9611082; doi:10.3390/polym14204411)
Supplement: Supplementary file 1 [file polymers-14-04411-s001.zip › polymers-1880888-supplementary.pdf]

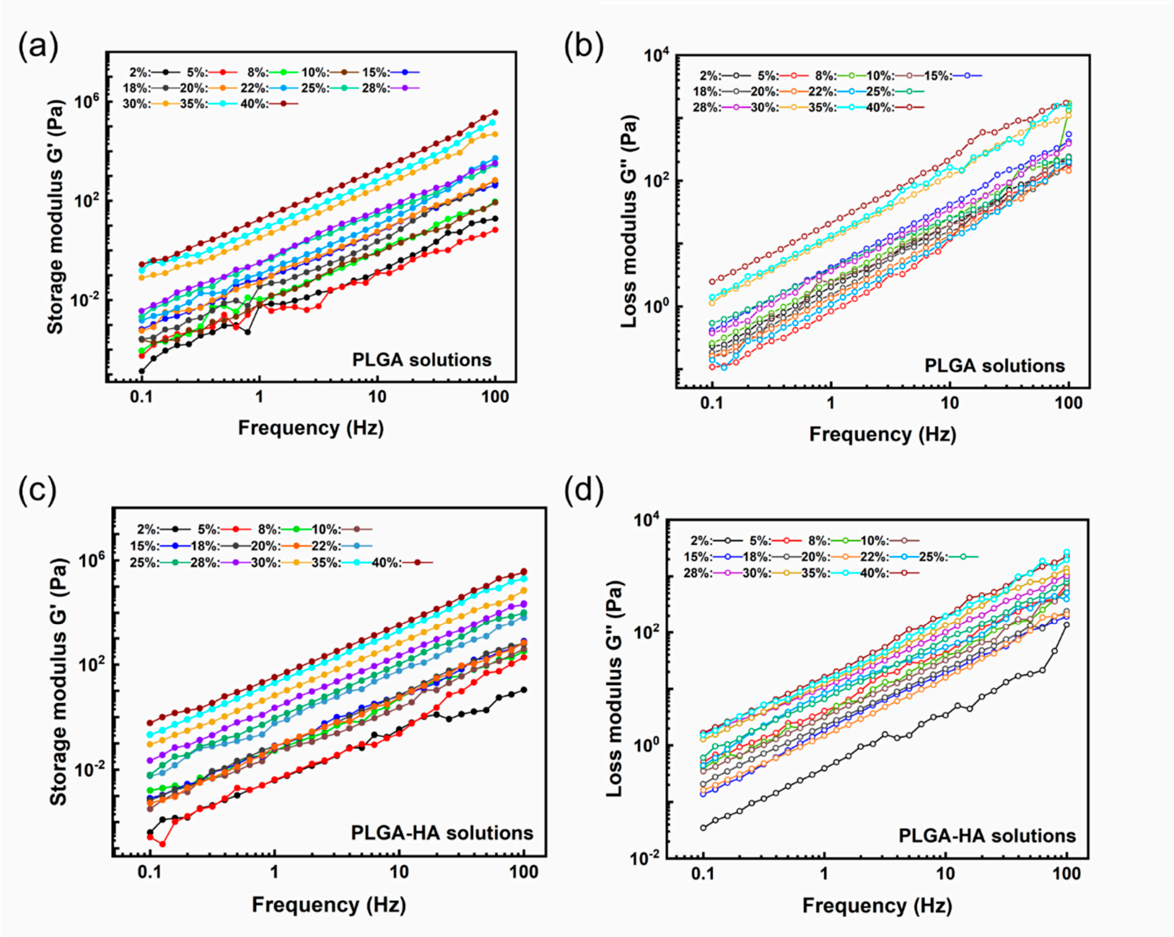

Figure S1. Complex moduli for PLGA and PLGA-HA solutions. Generally, higher PLGA concentration shows higher module values.

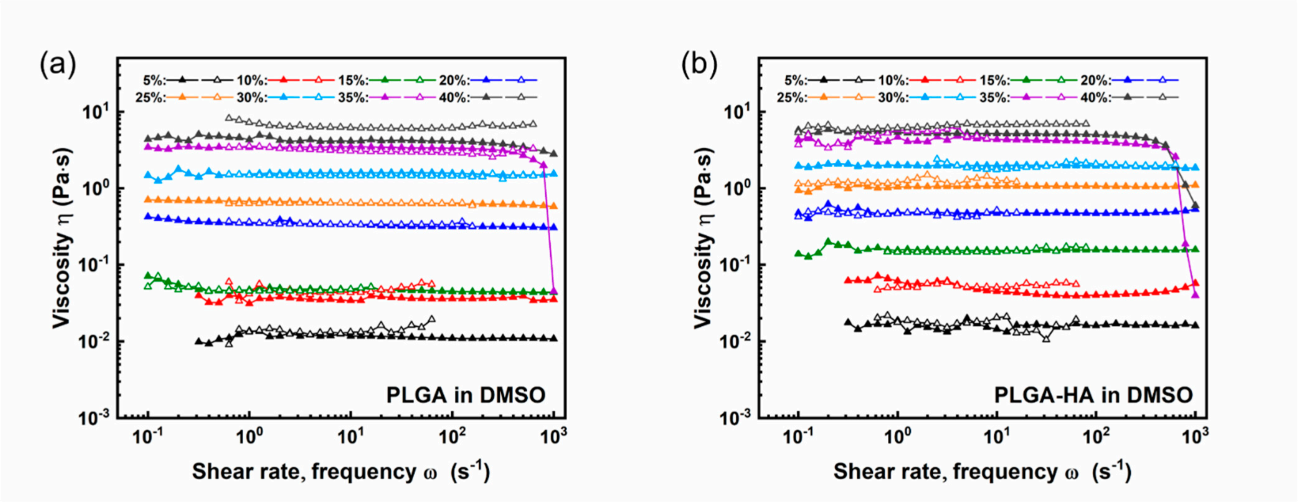

Figure S2. Cox-Merz relationship between steady shear viscosity (filled symbols) and complex viscosity from oscillatory experiments (open symbols) and for PLGA and PLGA-HA solutions of selected concentrations (5%, 10% ,15%, 20%, 25%,30%, 35% and 40%).

Notes: The Cox-Merz rule is found to work empirically for solutions and melts of many unlinked and unfilled polymers as $\eta(\dot{\gamma}) = |\eta^*(\omega)|$. *Which means the shear rate-dependent viscosity determined by rotational tests and the angular frequency-dependent complex viscosity by oscillatory tests show an identical shape of the curve over a wide range. In other words,* η *and* η^* *exhibits the same value. It is also reported that complex viscosity indicates the zero-shear viscosity at low* ω *values*[1]. *Eight concentrations from both semi-dilute unentangled and semi-dilute entangled regimes were selected to test the rule. And the Cox-Merz rule was well validated.*

(ref:1. Mezger, T.G. *The Rheology Handbook: For Users of Rotational and Oscillatory Rheometers*; 4th ed.; Vincentz Network: Hanover, Germany, 2014; ISBN 9783866306509.)

Table S1. Statistical analysis of solution conductivity results with p-values investigated.
*p<0.001: ***, p<0.01: **, p< 0.05: **

Concentration of PLGA in different polymer solutions		Pure PLGA solutions					PLGA-HA solutions			
		20%	25 %	30 %	35 %	40 %	25%-1.5%	30%-1.5%	35%-1.5%	40%-1.5%
Pure PLGA solutions	20 %		*** 6.85E-07	*** 3.55E-10	*** 5.84E-11	*** 4.38E-10				
	25 %			*** 4.42E-06	*** 1.73E-06	*** 3.14E-07				
	30 %				0.40935	*** 1.84E-04				
	35 %					*** 2.52E-04				
PLGA-HA solutions	20 %-1.5 %	*** 3.33E-14					*** 2.07E-04	*** 9.17E-07	*** 2.72E-05	*** 3.98E-05
	25 %-1.5 %		*** 6.44E-10					0.4041	0.65101	* 0.03808
	30 %-1.5 %			*** 1.85E-10					0.06026	*** 3.16E-05
	35 %-1.5 %				*** 3.04E-10					* 0.01384
	40 %-1.5 %					*** 4.13E-08				

Table S2. Statistical analysis of solution surface tension results with p-values investigated.
*p<0.001: ***, p<0.01: **, p< 0.05: **

Concentration of PLGA in different polymer solutions		Pure PLGA solutions					PLGA-HA solutions			
		20%	25 %	30 %	35 %	40 %	25%-1.5%	30%-1.5%	35%-1.5%	40%-1.5%
Pure PLGA solutions	20 %		0.66857	*** 1.82E-04	*** 7.87E-04	*** 8.08E-05				
	25 %			*** 1.93E-05	*** 1.95E-04	*** 1.19E-04				
	30 %				0.97617	** 0.00943				
	35 %					* 0.01511				
PLGA-HA solutions	20 %-1.5 %	** 4.64E-03					0.77378	* 0.03003	*** 3.32E-06	*** 6.31E-4
	25 %-1.5 %		** 0.00204					* 0.08479	*** 4.13E-05	*** 7.85E-4
	30 %-1.5 %			* 0.0245					*** 1.34E-04	** 0.00203
	35 %-1.5 %				* 0.03892					0.12617
	40 %-1.5 %					0.82627				

Table S3. Statistical analysis of diameter of electrospun nanofibres with p-values investigated.
*$p<0.001$: ***, $p<0.01$: **, $p< 0.05$: **

Concentration of PLGA in different polymer solutions		Pure PLGA solutions					PLGA-HA solutions			
		20%	25 %	30 %	35 %	40 %	25%-1.5%	30%-1.5%	35%-1.5%	40%-1.5%
Pure PLGA solutions	20 %		*** 0	*** 0	*** 0	*** 0				
	25 %			** 0.00961	*** 0	*** 0				
	30 %				*** 3.00E-15	*** 0				
	35 %					* 0.03972				
PLGA-HA solutions	20 %-1.5 %	*** 0					0.28556	*** 8.39E-6	*** 0	*** 0
	25 %-1.5 %		*** 0					*** 5.43E-4	*** 0	*** 0
	30 %-1.5 %			*** 0					*** 0	** 0.00203
	35 %-1.5 %				*** 0					*** 1.27E-5
	40 %-1.5 %					*** 0				
